# Timing of cesarean section for prolonged labor in urban Tanzania: A criterion-based audit

**DOI:** 10.1016/j.xagr.2024.100404

**Published:** 2024-10-10

**Authors:** Monica Lauridsen Kujabi, Natasha Housseine, Idrissa Kabanda, Rukia Msumi, Luzango Maembe, Mtingele Sangalala, Manyanga Hudson, Sarah Hansen, Anna Macha, Brenda Sequeira D'mello, Dan Wolf Meyrowitsch, Flemming Konradsen, Andreas Kryger Jensen, Kidanto Hussein, Nanna Maaløe, Thomas van den Akker

**Affiliations:** 1 Global Health Section, Department of Public Health, University of Copenhagen, Copenhagen, Denmark (Kujabi, Housseine, D'mello, Meyrowitsch, Konradsen, and Maaløe); 2 Department of Obstetrics and Gynaecology, Regional Hospital Randers, Randers, Denmark (Kujabi); 3 Medical College East Africa, Aga Khan University, Dar es Salaam, Tanzania (Housseine, Macha, D'mello, and Hussein); 4 Municipal Hospitals of Ubungo (Sinza) and Temeke (Mbagala Rangitatu), Presidents Office, Regional and Local Government, Dar es Salaam, Tanzania (Kabanda and Msumi); 5 Regional Referral Hospital (Temeke/Amana/Mwananyamala), Ministry of Health, Community Development, Gender, Elderly and Children, Dar es Salaam, Tanzania (Maembe, Sangalala, and Hudson); 6 Department of Clinical Medicine, Aarhus University, Aarhus, Denmark (Hansen); 7 Comprehensive Community Based Rehabilitation Tanzania Hospital, Dar es Salaam, Tanzania (D'mello); 8 Novo Nordisk Foundation, Hellerup, Denmark (Konradsen); 9 Section of Biostatistics, Department of Public Health, University of Copenhagen, Copenhagen, Denmark (Jensen); 10 Department of Obstetrics and Gynaecology, Copenhagen University Hospital Hvidovre, Hvidovre, Denmark (Maaløe); 11 Athena Institute, Vrije Universiteit Amsterdam, Amsterdam, The Netherlands (van den Akker); 12 Department of Obstetrics and Gynaecology, Leiden University Medical Center, Leiden, The Netherlands (van den Akker).

**Keywords:** childbirth, dystocia, guidelines, low-resource setting, PartoMa, pregnancy, Tanzania, urban

## Abstract

**BACKGROUND:**

Similar to many resource-constrained urban settings, cesarean deliveries in Dar es Salaam, Tanzania, have increased rapidly, from 17% in 2015 to 26% in 2022. Alarmingly, at the population level, the increase was not followed by improvements in perinatal outcomes, suggesting the overuse of cesarean delivery. Prolonged labor is the leading cause of women's first cesarean delivery. Therefore, understanding the management of prolonged labor preceding cesarean delivery is crucial for preventing nonmedically indicated cesarean deliveries across Tanzania and globally.

**OBJECTIVE:**

This study aimed to estimate the proportion of cesarean deliveries with a written indication of prolonged labor that was performed in labors with uncomplicated progression.

**STUDY DESIGN:**

This study was conducted at 5 urban maternity units in Dar es Salaam, Tanzania, from October 1, 2021, to August 31, 2022. Data were extracted from case files of women who gave birth via cesarean delivery with a written indication of prolonged labor. The timing of cesarean delivery decision was assessed against predefined definitions of prolonged labor at each stage/phase of labor. The proportion of cesarean deliveries performed in cases of uncomplicated progression was calculated. The exclusion criteria included referral to study sites because of prolonged labor or cervical dilatation of >6 cm upon admission, noncephalic presentation, multiple pregnancy, intrauterine fetal death, failed induction of labor, previous cesarean delivery, or other written indications for cesarean delivery.

**RESULTS:**

The overall cesarean delivery rate was 32% (2949/9364). Of first-time cesarean delivery cases, 746 of 1517 patients (47.9%) had a written indication of prolonged labor. Finally, 456 of 746 patients (61.1%) met the inclusion criteria, of which 307 of 456 patients (67.3%) were admitted in the latent phase of labor. In 243 of 456 cesarean deliveries (53.3%) with an indication of prolonged labor, labor was not prolonged. This group included (1) women not being given a trial of labor (78/243 [32.1%]), (2) women in the first stage of active labor not crossing the partograph action line (145/243 [59.7%]), and (3) women in the second stage of labor lasting <1 hour (20/243 [8.2%]). Of note, 78 of 346 women (21.5%) in the first stage of active labor had a labor progression faster than 0.5 cm per hour preceding the decision for cesarean delivery.

**CONCLUSION:**

Almost half of cesarean deliveries in unscarred uteri were because of prolonged labor. Despite a written indication of prolonged labor, approximately half of the cases did not have prolonged labor. Although care in low-resource settings has traditionally been categorized as “too little, too late,” this study finds care as “too much, too soon” in one of the world's fastest-growing urban areas. This finding highlights the inadequacy of one-size-fits-all approaches in curbing the increases in cesarean delivery occurring in (pockets of) low-resource settings. Our study calls for ways to respectfully allow more time for physiological labor progression in busy high-volume maternity units where many births occur.

**AJOG Global Reports at a Glance**

**Why was this study conducted?**

Similar to many resource-constrained urban settings, cesarean deliveries (CDs) in Dar es Salaam are increasing rapidly, not accompanied by a decrease in perinatal deaths. Little focus has been on ensuring timely management of labor progression in urban high-volume understaffed maternity units.

**Key findings**

More than half of CDs with the reported indication of prolonged labor were performed with uncomplicated progress. Outdated definitions of prolonged labor were used, and the resource-constrained context seemed to induce defensive decision-making.

**What does this add to what is known?**

Focus in low-resource settings is often on delayed care and unmet needs. In contrast, our study found that CDs were often performed too early. Our results indicate that there is a crucial need to explore how to respectfully allow more time for physiological labor progression in urban high-volume low-resource maternity units.

## Introduction

Although cesarean deliveries (CDs) can be lifesaving, the latest decades’ dramatic increase in CDs globally is not accompanied by a simultaneous decrease in adverse perinatal outcomes.^1^ This raises concern about dangerous overuse.^2^^,^^3^ At current speed, the global CD rate is predicted to increase from 21.1% of all births to date to 28.5% (38 million CDs) in 2030. Of these CDs, 88% will occur in low- and middle-income countries where CD-related risks are highest and where cost-efficient healthcare is needed.^3^^,^^4^

Compared with vaginal birth, CD is related to increased maternal and perinatal risks.^5^ In sub-Saharan Africa, women face a 1% risk of maternal death associated with CD.^3^ In addition, the risk of uterine rupture and placenta accrete spectrum disorders in subsequent pregnancies may be fatal.^6^ CDs are costly, and the human and material resources spent on unnecessary CDs may indirectly cost lives elsewhere.^7^ Meanwhile, many women in the poorest parts of the world still suffer from a delay in receiving an indicated CD.^4^ Consequently, ending the current “CD pandemic,” which harms women and children, must be a global health priority.

Prolonged labor is the leading cause of CD in the unscarred uteri and a crucial driver of the increasing CD rates.^1^^,^^4^ Between one-third to one-fourth of all unplanned CDs are performed for prolonged labour.^8^ A key contributor may be substandard diagnosis and management of prolonged labor. Studies show overuse of CDs performed when the membranes were still intact, oxytocin augmentation not applied, or without second-stage vacuum extraction attempted.^9^, ^10^, ^11^, ^12^ This is in contrast to our experience in Dar es Salaam, where data indicated the use of oxytocin to augment labor despite uncomplicated labor progression.^13^

In Tanzania, data suggest a trend toward growing urban disadvantages in maternal and perinatal health.^14^^,^^15^ In Dar es Salaam, facility-based maternal and neonatal mortality rates have stagnated. In a study by Sequeira Dmello et al,^16^ maternal mortality was 82 per 100,000 live births in 2015 and 80 per 100,000 live births in 2019, and neonatal mortality was 14.5 per 1000 live births in 2014 and 12.1 per 1000 live births in 2019. Meanwhile, CDs have increased from 17% in 2015 to 26% in 2022.^17^^,^^18^ The World Health Organization (WHO) recommends a population CD rate of 10% to 15%. This indicates that maternal health programs in cities are not meeting the needs.^19^ Consequently, as part of the PartoMa project for reaching locally useful clinical practice guidelines (CPGs) in 5 of the most congested governmental maternity units in Dar Es Salaam, Tanzania, we conducted an in-depth criteria-based audit to explore the proportion of CD with a written indication of prolonged labor despite uncomplicated labor progression.

## Materials and methods

This study was conducted at 5 governmental maternity units in Dar Es Salaam, Tanzania, with an annual number of births ranging from 7000 to 14,000.^20^, ^21^, ^22^ The 5 governmental maternity units are referral sites providing comprehensive basic and emergency obstetrical care to low- and high-risk women, including CDs, around the clock. Women wait a median of 2.3 hours for an unplanned CD. Nurse-midwives provide care during labor. The nurse-midwife–to–laboring women ratio range from 1:1 to 1:7. The midwives’ tasks include triaging women, providing immediate postnatal care, escorting women for referral, filling stocks, and cleaning. Medical doctors decide on CD and oxytocin augmentation. In the absence of a doctor, a nurse-midwife makes an independent decision or consults a doctor by phone. The partograph is used to monitor labor and fetal heart rate (FHR), is monitored by intermittent Pinard auscultation or handheld Doppler. Birth companions are not allowed, and epidural analgesia is not available. Vacuum extractors are available. However, not all clinicians are competent/confident to perform the procedure. Consequently, the rate of vacuum births at the 5 maternity units is approximately 3%. National guidelines exist on the dose of oxytocin augmentation. The partograph guide the timing of oxytocin for labor augmentation but is not standardized across hospitals. As explored by Kujabi et al^13^ at one of the maternity units, oxytocin to augment labor is used at high rates to hasten labor progression and is influenced by the high volume of women and lack of beds. More details about the study sites and the PartoMa project, which the study is part of, are published elsewhere.^20^^,^^21^

### Development of locally achievable evidence-based audit criteria

The audit criteria were developed from the PartoMa CPGs for childbirth care. The PartoMa CPGs were based on global evidence-based recommendations, cocreated with 107 Tanzanian health providers, peer-reviewed by an international panel of experts, and approved by the Tanzanian Ministry of Health. This enabled the audit criteria to reflect standards on the best possible evidence-informed care when taking local resources into account.^23^

**Figure 1:**
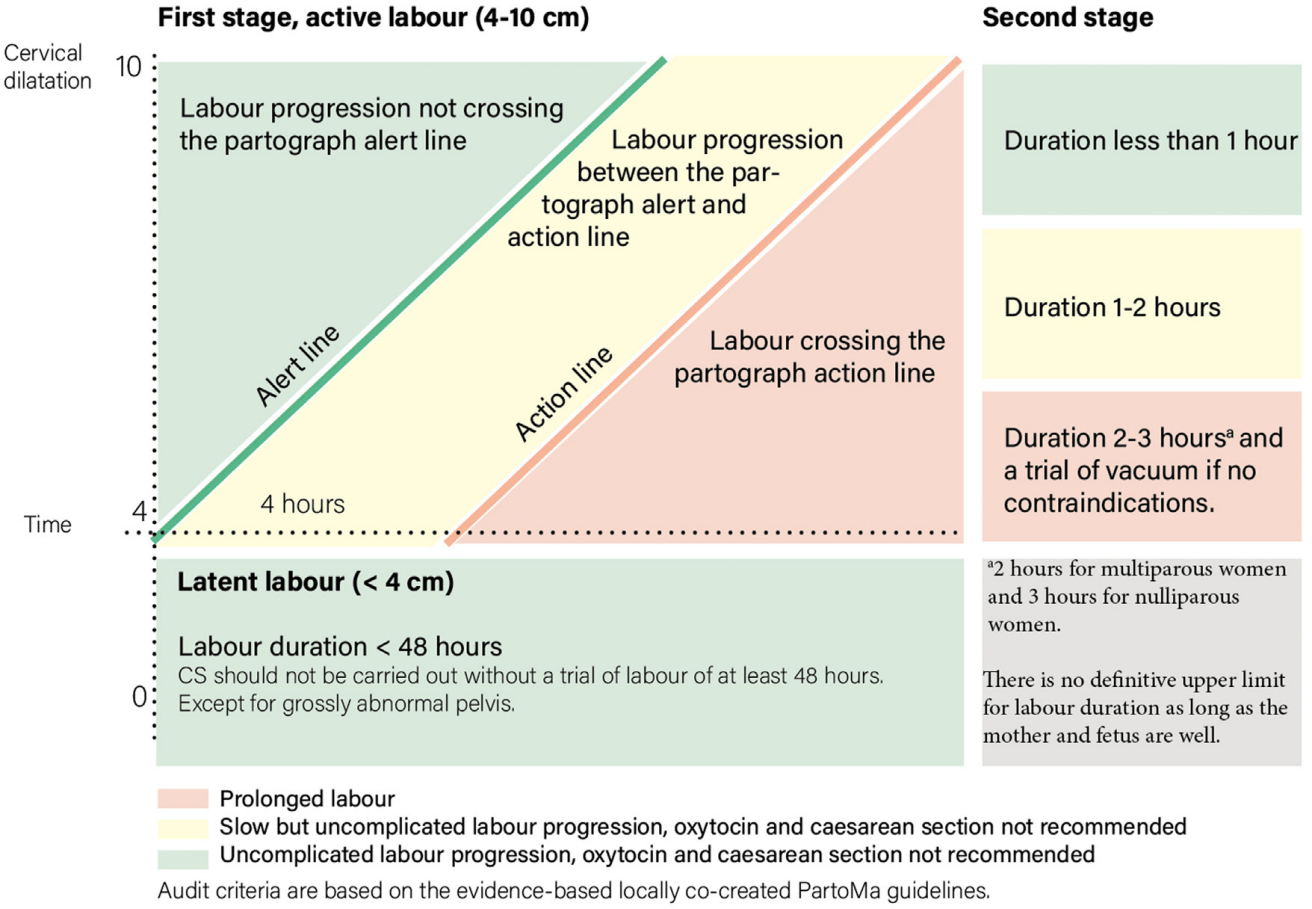
Audit criteria used to evaluate CD because of prolonged labor *CD*, cesarean delivery.

As shown in Figure 1, the audit criteria were made for each stage/phase of labor, and except for second stage of labor, the same criteria were applied for nulliparous and multiparous women. The criteria categorized women into 3 groups: (1) women having CD during normally progressing labor, including women who were never given a trial of labor (green); (2) women having CD during slow labor progression (yellow); and (3) women having CD during prolonged labor (red). CDs decided in categories 1 and 2 can be considered potentially avoidable. The PartoMa CPGs and background information are available online.^24^

Kujabi. Cesarean section for prolonged labor in Tanzania. Am J Obstet Gynecol Glob Rep 2024.

### Data collection

Data collection took place between October 1, 2021, and August 31, 2022, during a 3-month period at each study site.^20^^,^^21^ The study population included all women who gave birth via CD with an indication of prolonged labor written in the case file.

As terms were used interchangeably, we use prolonged labor as an “umbrella indication,” which includes “prolonged labor,” “poor progress of labor,” “failure of augmentation,” “cervical arrest,” “cephalopelvic disproportion (CPD),” “big baby,” and “obstructed labor.”

We excluded referred women with cervical dilatation >6 cm upon admission or in whom the referral diagnosis was prolonged labor, non-cephalic pregnancy, multiple pregnancy, intrauterine fetal death, failed induction of labor, and previous CD. Furthermore, women were excluded if they had more than 1 indication for CD and the other indication (in addition to prolonged labor) could also be a reason for CD (ie, placental abruption, fetal distress, and chorioamnionitis). By using strict inclusion/exclusion criteria, we aimed to identify CDs that were not influenced by other factors than prolonged labor.

All case files were collected from the hospital storage after discharge. Case files were cross-checked with birth registry books (Mfumo wa Taarifa za Uendeshaji Huduma za Afya). All births were entered to provide the overall CD rate, and CDs due to other indications were analyzed elsewhere (not yet published). CDs with prolonged labor as a written indication in the case file were identified. For these CDs, data from birth registries and case files were reviewed and double-entered into prepiloted forms using the KoBoToolbox. Data were collected and entered by research assistants with medical backgrounds. Data collectors did not make interpretations. In case of uncertainty (eg, difficult handwriting), the hospital staff (M.L.K. or N.M.) was consulted.

Validation of the data in case files was performed before the study and showed that the concordance between performed practices and the medical records was >95% for vaginal examinations, times, and background characteristics.

### Variables, analysis, and audit

Descriptive statistics were performed using R (version 4.1.2; R Foundation for Statistical Computing, Vienna Austria; November 1, 2021). Labor progression curves were created by plotting women's first cervical dilatation of ≥4 cm on the partograph alert line and inserting each progression (time on the x-axis and cervical dilatation on the y-axis) after this until CD decision. Background characteristics included hospital, age, gestational age, parity, referral status, stage of labor, maternal complications, perinatal outcomes, and birthweight. Intrapartum management included information on labor duration, labor progression, and labor interventions (induction, amniotomy, and oxytocin). The dose and duration of oxytocin were not available in the case files. Unfortunately, data on pushing/exhaustion, pain, contractions, mobilization, and fluid consumption were not available in the case files.

CDs were evaluated on the basis of labor stage: (1) before active labor (cervical dilatation of <4 cm), (2) first stage of active labor (cervical dilatation of 4 to <10 cm), and (3) second stage of labor (10 cm/full dilatation). If the partograph was not used, the data collectors plotted cervical measurements on a partograph to visualize labor progress. The audit was conducted by comparing the data entered against the audit criteria (Figure 1). This was performed by M.L.K. after all data were collected. CDs were grouped into the aforementioned categories (Figure 1). The proportions of each group were calculated. After analysis, the findings were discussed with clinical representatives from the 5 study hospitals to include their perspectives.

## Results

**Figure 2:**
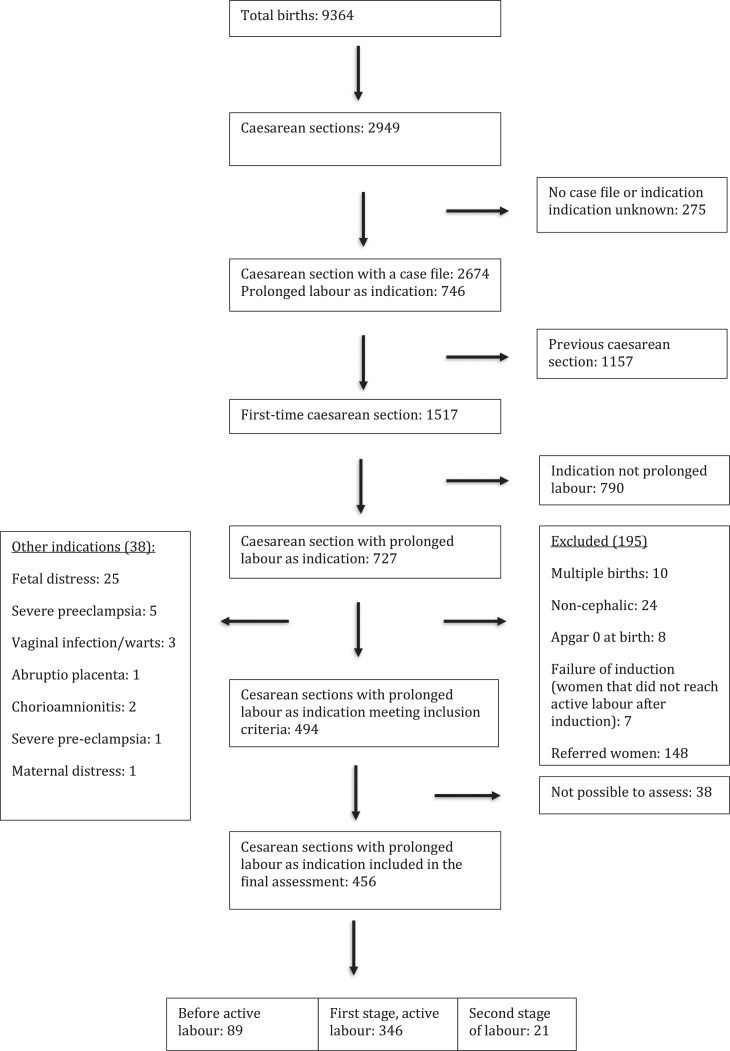
Flowchart

The combined CD rate at the 5 hospitals was 31.5% (2949/9364). Of these CDs, 788/2949 (26.7%) had a written indication of prolonged labor, accounting for 727/1517 CDs (47.9%) in unscarred uteri (Figure 2).

Kujabi. Cesarean section for prolonged labor in Tanzania. Am J Obstet Gynecol Glob Rep 2024.

The findings did not differ significantly among the hospitals, and combined analyses were presented. After excluding women not meeting the inclusion criteria, 456 women remained for analysis. Nulliparous women accounted for 277 of 456 CD cases (60.9%) (unknown = 1). Apgar scores were between 8 and 10 at 5 minutes in 454 of 456 patients (99.6%). The remaining background and labor characteristics are shown in Table 1.

**Table 1:** CD with a written indication of prolonged labor in 456 low-risk women

Background information	N=456
Referred	40 (8.8%)
Age (y), median (IQR)	25 (21–30)
Parity	
Nulliparous	277 (60.9%)
Multiparous without previous CD	178 (39.1%)
Unknown	1
Gestational age (wk)	
<37	13 (3.0%)
≥37	425 (97.0%)
Unknown	18
Birthweight (kg)	
0.0–2.4	10 (2.2%)
2.5–4.0	396 (86.8%)
>4.0	50 (11.0%)
Maternal complications (infection, cardiomyopathy, and bleeding of >1000 mL)	7 (1.5%)
Apgar score	
4–7	2 (0.4%)
8–10	454 (99.6%)
**Labor characteristics**	
Labor interventions	
Induction	71 (15.6%)
Oxytocin augmentation	138 (30.3%)
Status of labor on admission	
Not in labor	33 (7.2%)
Latent phase	274 (60.1%)
First stage	130 (28.5%)
Second stage	2 (0.4%)
In labor, stage/phase unknown	17 (3.7%)
Time between admission and CD decision (h)	
≤12	189 (43.9%)
>12	242 (56.1%)
Unknown	25
Time between decision and birth (h)	
≤2	199 (46.2%)
>2	232 (53.8%)

*CD*, cesarean delivery; *IQR*, interquartile range.

### Phase of labor at time of cesarean section decision

Women were divided into 3 groups: (1) before active labor (89/456 [19.5%]), (2) first stage of active labor (346/456 [75.9%]), and (3) second stage of labor (21/456 [4.6%]) (Table 2).

**Table 2:** CD with a written indication of prolonged labor: labor management according to the stages of labor at the time of CD decision

Variable	n (%)
**Women who had not reached active labor**	n=89
Time between admission and decision (h)	
0.0–23.9	59 (66.3%)
24.0–47.9	19 (21.3%)
48.0–96.0	11 (12.4%)
Birthweight (kg)	
2.5–3.9	76 (86.4%)
4.0–6.0	12 (13.6%)
Unknown	1
Grossly abnormal pelvis (hip joint deformity)	1 (1.1%)
Interventions	
Induction	10 (11.2%)
Membranes ruptured (spontaneously or artificially)	14 (16.8%)
Oxytocin augmentation	0 (0%)
**Women who were in the first stage of active labor**	n=346
Progress of labor at the time of decision	
On or before crossing the alert line	53 (15.3%)
Between the alert line and action line	92 (26.6%)
On or after crossing the action line	201 (58.1%)
Cervical progression between the last 2 vaginal examinations (cm/h)	
0.0	138 (51.1%)
0.1–0.5	74 (27.4%)
>0.5	58 (21.5%)
Unknown	76
4 h of labor arrest	79 (22.8%)
Interventions	
Membranes ruptured (spontaneously or artificially)	308 (89.0%)
Oxytocin augmentation	132 (38.2%)
Progress of labor at the time oxytocin was prescribed (of 132 augmented)	
On or before crossing the alert line	65 (52.8%)
Between the alert line and action line	36 (29.3%)
On or after crossing the action line	22 (17.9%)
Unknown	9
**Women who were in the second stage of labor**	n=21
Duration of the second stage (h)	
≤1	20 (95.2%)
≥2	1 (4.8%)
Attempted vacuum-assisted birth	
Yes	1 (4.8%)
No	20 (95.2%)

*CD*, cesarean delivery.

**Figure 3:**
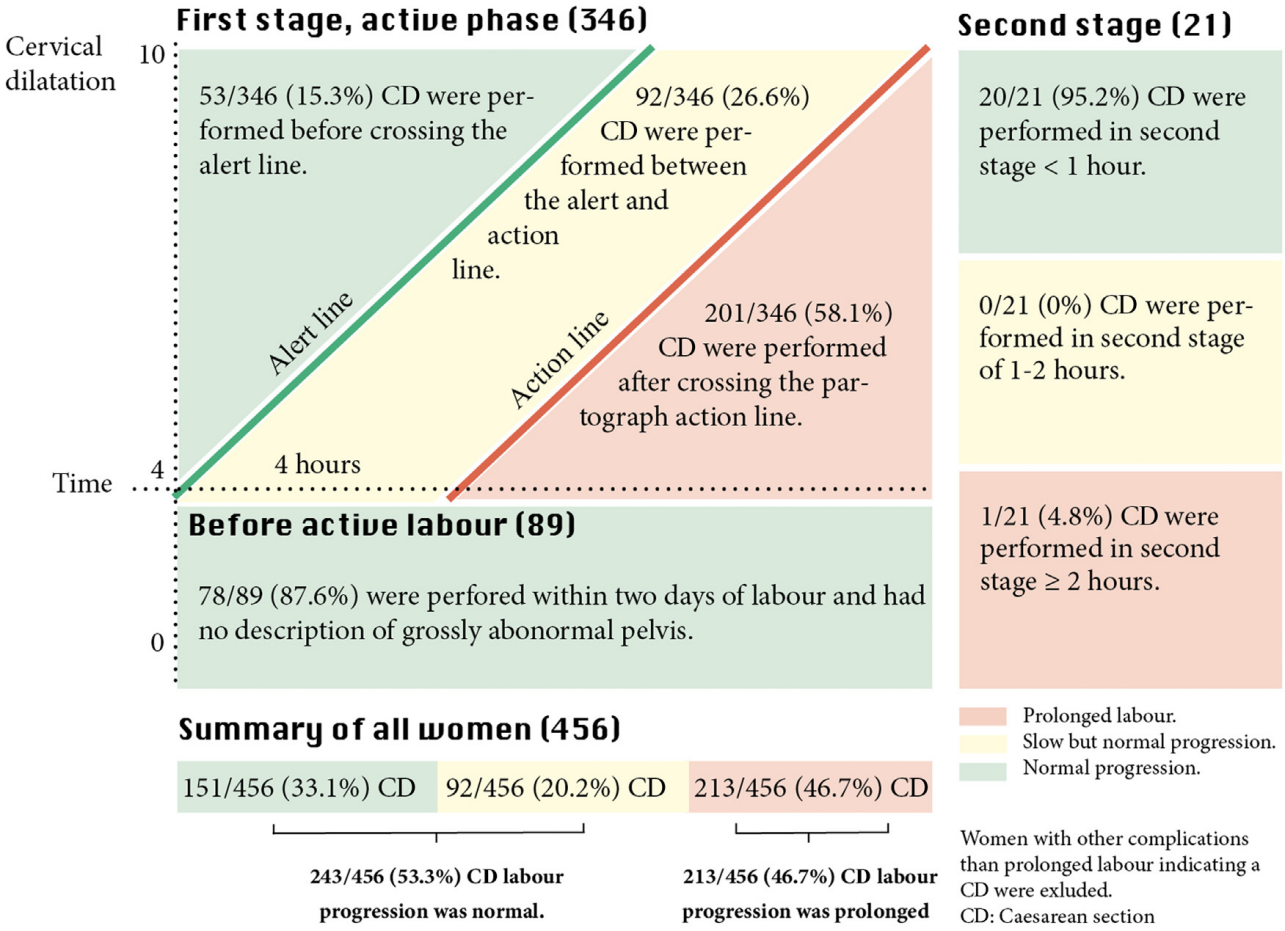
Evaluation of CD because of prolonged labour in 456 women Women are divided into stage/phase of labor at the time of CD decision. *CD*, cesarean delivery.

In 89 of 456 women (19.5%) with a CD decision before active labor, 78 of 89 women (87.6%) were not given a trial of labor (Figure 3). Other characteristics of this group can be seen in Table 2.

Kujabi. Cesarean section for prolonged labor in Tanzania. Am J Obstet Gynecol Glob Rep 2024.

**Figure 4:**
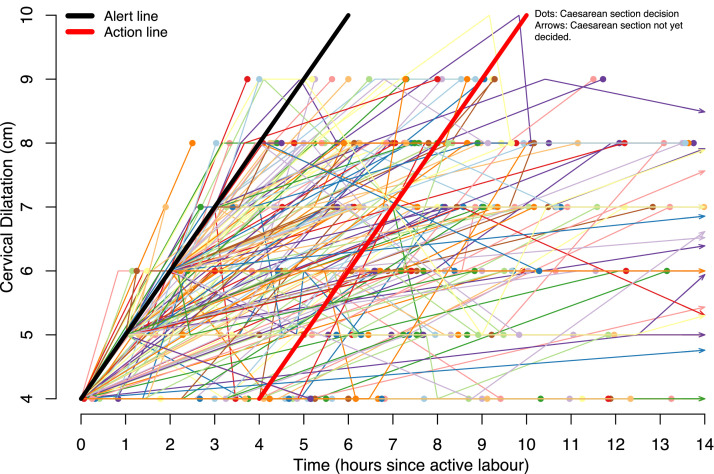
CD during first stage active labour: Individual labor progression curves

In 346 of 456 women (75.9%) with CD decided during the first stage of active labor, 145 of 346 CDs (41.9%) were performed before crossing the partograph action line. In addition, 53 of 346 CDs (15.3%) did not cross the alert line, and 92 of 346 CDs (26.6%) were between the alert and action lines (Figure 3). This is apparent when looking at Figure 4, which shows individual progression curves. Figure 4 is split into 3 subfigures (Supplementary Material) to visualize labor progression and duration in subgroups of women (Figure S1A: women who did not cross the action line; Figure S1B: women who crossed the action line; Figure S1C: women with a cervical progression of >0.5 cm/h preceding CD decision). More details on labor progression and timing of oxytocin augmentation preceding CD decision are presented in Table 2.

Kujabi. Cesarean section for prolonged labor in Tanzania. Am J Obstet Gynecol Glob Rep 2024.

In 21 of 456 women (4.6%) with CD decided during the second stage of labor, 20 of 21 CDs (95.2%) were performed when the duration of the second stage of labor had lasted <1 hour (Figure 3). Vacuum extraction was attempted in 1 of 21 woman (4.8%) (Table 2).

### Cesarean delivery in uncomplicated labor progression

Overall, 243 of 456 CDs (53.3%) did not meet the audit criteria because these were decided despite uncomplicated labor progression (Figure 3). Figure 3 shows the distribution according to the stage/phase of labor.

### Use of diagnostic terms

As shown in Table 3, many different terms for prolonged labor were used (obstructed labor (35.1%), big baby (26.1%), prolonged labor/poor progress (25.9%), cervical arrest (17.1%), and CPD (13.8%). Some women had more than 1 diagnosis. Descriptions used for each term are presented in Table 3. For example, of 160 women with “obstructed labor,” only 51 (31.8%) had cervical arrest, and 38 (24.4%) had nothing described. Of women with “cervical arrest” as indication, half did not have cervical arrest according to the written vaginal examinations, and of women with “big baby” as indication, only 27.7% had a birth weight >4 kg.

**Table 3:** Terms used to diagnose prolonged labor and associated clinical findings

Terms	N=456
Obstructed labor	160 (35.1%)
Cervical arrest between the last 2 vaginal examinations	51 (31.9%)
Head 1/5 or more palpable per abdomen	46 (28.8%)
Station above the ischial spines (−1, −2, −3)	4 (2.5%)
No description	39 (24.4%)
Big baby	119 (26.1%)
Birthweight of ≥4 kg	33 (27.7%)
Prolonged labor/poor progress	118 (25.9%)
The latest cervical progression slower than 0.5 cm/h	87 (73.7%)
Cervical arrest	78 (17.1%)
No progression between the last 2 vaginal examinations	39 (50.0%)
No progression within the last 4 h	27 (34.6%)
CPD	63 (13.8%)
Borderline/inadequate pelvis	9 (14.3%)
Prominent pubic angle/unfavorable pelvimetry	2 (3.2%)
Others^a^	4 (6.3%)
No description	48 (76.2%)

*CPD*, cephalopelvic disproportion.

a Swollen cervix, contracted pelvis, hip joint deformity, and maternal heigh of 146 cm.

## Discussion

### Principal findings

At 5 of the busiest governmental maternity units in Dar es Salaam, Tanzania, prolonged labor accounted for 47.9% of CDs in unscarred uteri. This study revealed a wide gap between the latest evidence and clinical practice. Despite a written indication of prolonged labor, 53.3% of women did not have prolonged labor (eg, without a trial of labor, before crossing the partograph's action line, without arrested labor, or during the second stage of labor lasting <1 hour). Detailed multicenter inquiries into CDs at the frontline of congested urban maternity units are extremely scarce, and the observed findings strongly call for action on how to ensure women their physiological labor.

### Results

Untreated prolonged labor is a tragedy, particularly because of delays in seeking and receiving care.^25^^,^^26^ This is important in understanding why our findings of too-early diagnosis may represent fundamental health system challenges and coping strategies in constrained labor wards rather than simply seeing this as substandard clinical judgments as concluded in other studies.^9^, ^10^, ^11^, ^12^ This study, combined with conversations with the staff, crucially clarifies how fear around prolonged labor, the inability to ensure fetal monitoring, and long waiting time for CD may be strong facilitators of defensive decision-making. As obstructed labor is a feared complication, unpublished conversations have revealed that health providers try to predict complications. Therefore, factors, such as previous intrauterine death, could influence defensive decisions. This may explain why CDs are decided before “real” prolonged labor. Defensive management may increase in countries where maternal and neonatal deaths are highly politicized and where giving birth is fraught with risks.^27^, ^28^, ^29^ Although particularly long-term CD risks are difficult to count, immediate good outcomes of CD, like in this study, can prevent blame and shame for health providers.

Women admitted in latent labor consist of more than half of the included women. It is well studied that admission in latent labor results in more interventions than later admission.^30^^,^^31^ However, a key strategy in many low-resource settings has been early admission for safe hospital birth, especially for women living in remote areas or facing heavy traffic jams.^32^^,^^33^ The latent phase of labor may last for many days, and performing a CD in latent labor/before labor is controversial.^1^ Care for women in latent labor can be resource draining if staff and beds are few.

Almost half of the women stayed <12 hours from admission to CD decision, and unpublished conversations revealed that 1 cm per hour was considered normal progression. This may represent relics from the Irish active management of labor (AML) strategy documented in the 1970s but not recommended anymore.^34^^,^^35^ This strategy focused on controlling the duration of labor to accommodate the increasing volume of births. Although studies have failed to document its effectiveness, the 1 cm per hour is still commonly used.^35^^,^^36^ Conversely, to date, no upper limit for labor exists, and the 1-cm-per-hour rule is not recommended.^1^ The latest evidence shows that labor progression is much slower, and this evidence is included in the recent WHO Labour Care Guide.^37^^,^^38^ In addition, it is discussed at the global level whether to start the partograph at 6 cm instead of 4, which could avert more CDs. The early diagnosis and overuse of CD in this study contradict this evidence. Similar practice is seen in many other places where outdated definitions are used and are observed to cause inappropriate treatment.^8^^,^^36^

Most CDs in the second stage of labor were decided within 1 hour of reaching the second stage of labor. Although international standards allocate up to 5 hours for nulliparous women in the second stage of labor, there is little evidence on the safety of this strategy in urban, overburdened, low-resource settings where FHR cannot be monitored closely and without immediate access to CD.^39^

### Clinical implications

**T**his study, like many others, finds an alarming gap between evidence and clinical practice. In addition, updated and translated clinical guidelines on labor progression are long overdue.^8^^,^^36^ However, it is crucial to look beyond the guidelines and training of healthcare providers and into the urban high-volume low-resource context.^13^ The message is clear: ensuring more time to labor will likely reduce CD. The high volume of women, combined with staff and bed shortages, may prevent the latest evidence on physiological labor progression from being integrated into clinical practice. This implies that political will and prioritization of women's health are necessary to ensure that labor wards are conducive for all phases of physiological birth. Moreover, defensive maternity care must be tackled, for example, through contextual evidence-based guidelines and CD audits supported by good clinical leadership.

### Research implications

It is evident that labor progresses much slower than previously defined, resulting in the WHO Labour Care Guide, in which uncomplicated active labor can take up to 18 hours with no intervention required. Although allowing more time is crucial in reducing CDs, we call for implementation research to investigate how such strategies can be implemented in high-volume, low-resource labor wards with limited monitoring. Such research must include balancing short- and long-term risks and benefits of allowing time vs potentially congested labor wards.

### Strengths and limitations

The strengths of this study include high-quality data with double entry of all case files. A limitation of this study is that the study was retrospective and that unwritten external factors possibly influencing care were not considered. Considering such factors is important as CD decision-making is complex, particularly in resource-constraint settings. Furthermore, what was not written was considered not done, which is not always the case. For example, oxytocin may have been underreported, and the rate must be interpreted with caution. The lack of differentiation between nulliparous and multiparous women reflects a gap in prolonged labor guidelines, which are more applicable to nulliparous women as multiparous women often progress substantially faster. Furthermore, at the hospitals, many terms for prolonged labor were used interchangeably, whereas none were explicitly defined by the hospitals. This represents a fundamental challenge in prolonged labor management and research globally.^36^

Many other studies have assessed CD decision by looking at correct management, such as amniotomy and oxytocin.^9^^,^^11^^,^^12^ Although this is also important in averting a CD, particularly in places where care is “too little, too late,” our study considers the timing factor, which appears crucial in high-volume settings characterized by “too much, too soon” care.^13^ Although caution should be taken to generalize findings from clinical audits, our findings are relevant to similar low-resource, high-volume labor wards.

## Conclusion

Many CDs were performed because of prolonged labor, Approximately half of CDs were performed in labor with uncomplicated labor progression. Efforts must be made to bridge the evidence-practice gap and move from AML to respecting and supporting woman's physiological progression. The complexity of urban low-resource labor wards where timely management is hampered by few staff and a continuous flow of women allows little time for physiological labor and the required monitoring. Therefore, indirectly, the unconducive labor ward becomes a central yet preventable driver of CDs.

## CRediT authorship contribution statement

**Monica Lauridsen Kujabi:** Writing – review & editing, Writing – original draft, Project administration, Methodology, Investigation, Funding acquisition, Formal analysis, Data curation, Conceptualization. **Natasha Housseine:** Writing – review & editing, Supervision, Project administration, Methodology, Conceptualization. **Idrissa Kabanda:** Writing – review & editing, Resources. **Rukia Msumi:** Writing – review & editing, Resources. **Luzango Maembe:** Writing – review & editing, Resources. **Mtingele Sangalala:** Writing – review & editing, Resources. **Manyanga Hudson:** Writing – review & editing, Resources. **Sarah Hansen:** Writing – review & editing, Resources, Project administration, Investigation, Formal analysis. **Anna Macha:** Writing – review & editing, Resources, Investigation. **Brenda Sequeira D'mello:** Writing – review & editing. **Dan Wolf Meyrowitsch:** Writing – review & editing, Funding acquisition. **Flemming Konradsen:** Writing – review & editing, Conceptualization. **Andreas Kryger Jensen:** Writing – review & editing, Formal analysis. **Kidanto Hussein:** Writing – review & editing. **Nanna Maaløe:** Writing – review & editing, Supervision, Methodology, Funding acquisition, Conceptualization. **Thomas van den Akker:** Writing – review & editing, Supervision, Methodology, Conceptualization.

## Appendix Supplementary materials

Image, application 1
