# Supplementary material for: Timing of cesarean section for prolonged labor in urban Tanzania: A criterion-based audit
Source: AJOG Glob Rep. 2024 Oct 10;4(4):100404. doi: 10.1016/j.xagr.2024.100404 (PMC11582455; doi:10.1016/j.xagr.2024.100404)

Supplementary material

Figure S1A-C: Individual labour progression curves were Figure 4 is split into S1A, S1B and S1C:

All figures show individual labour progression curves. The dots indicate the time when caesarean section was decided.

Figure S1A: Caesarean section decided before crossing the partograph's action line (114 women)

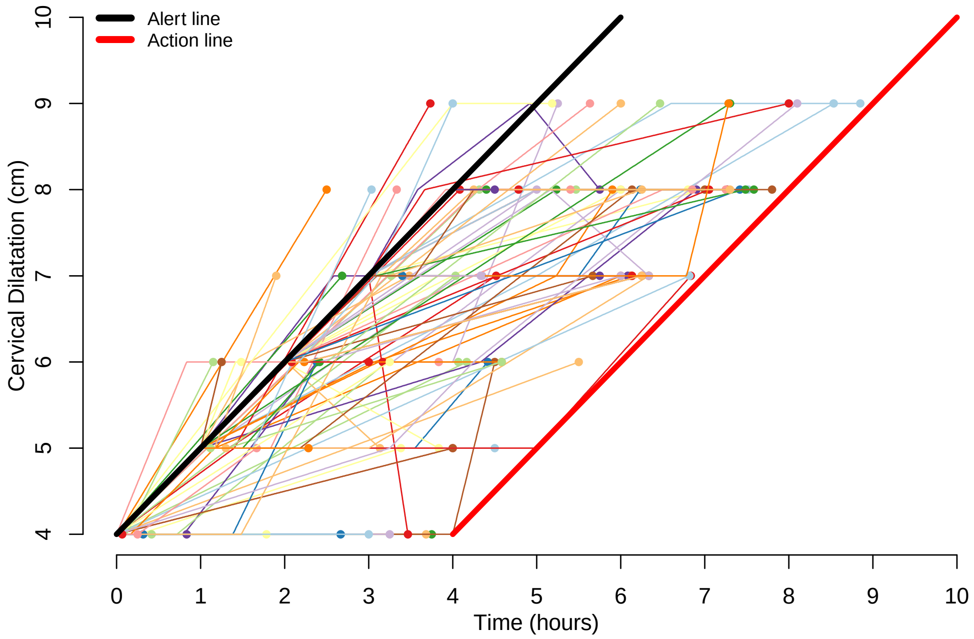

S1A shows that many of these women had a normal progression preceding caesarean section decision.

Figure S1B: Caesarean section decided after crossing the partograph's action line (196 women)

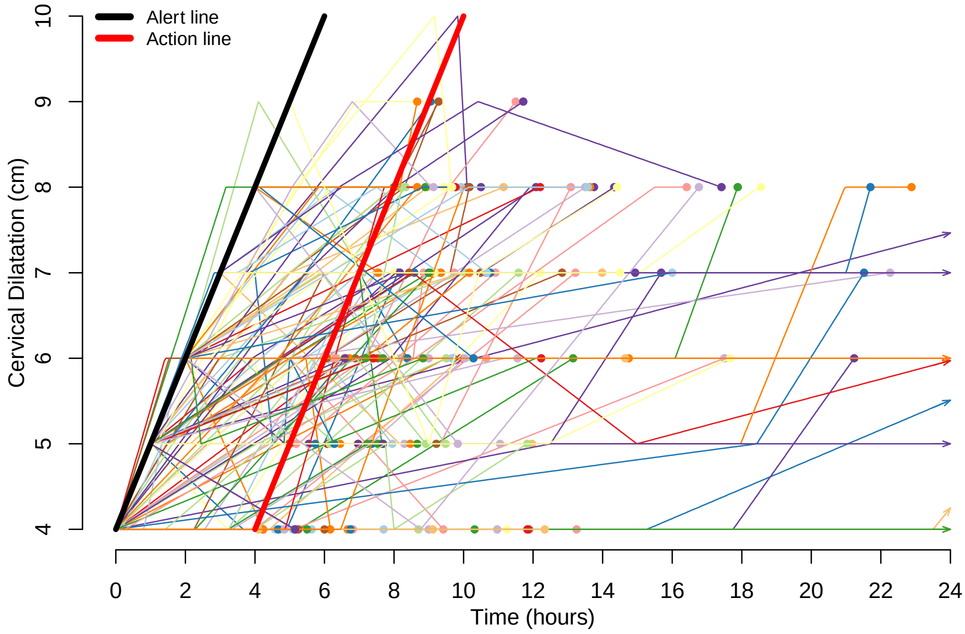

S2B further shows that some women had arrested labour for many hours before a caesarean section was decided, suggesting delayed diagnosis. The arrows means caesarean section was not yet decided.

Figure S1C: Women with labour progression > 0.5 cm/hour at the time caesarean section was decided (61 women)

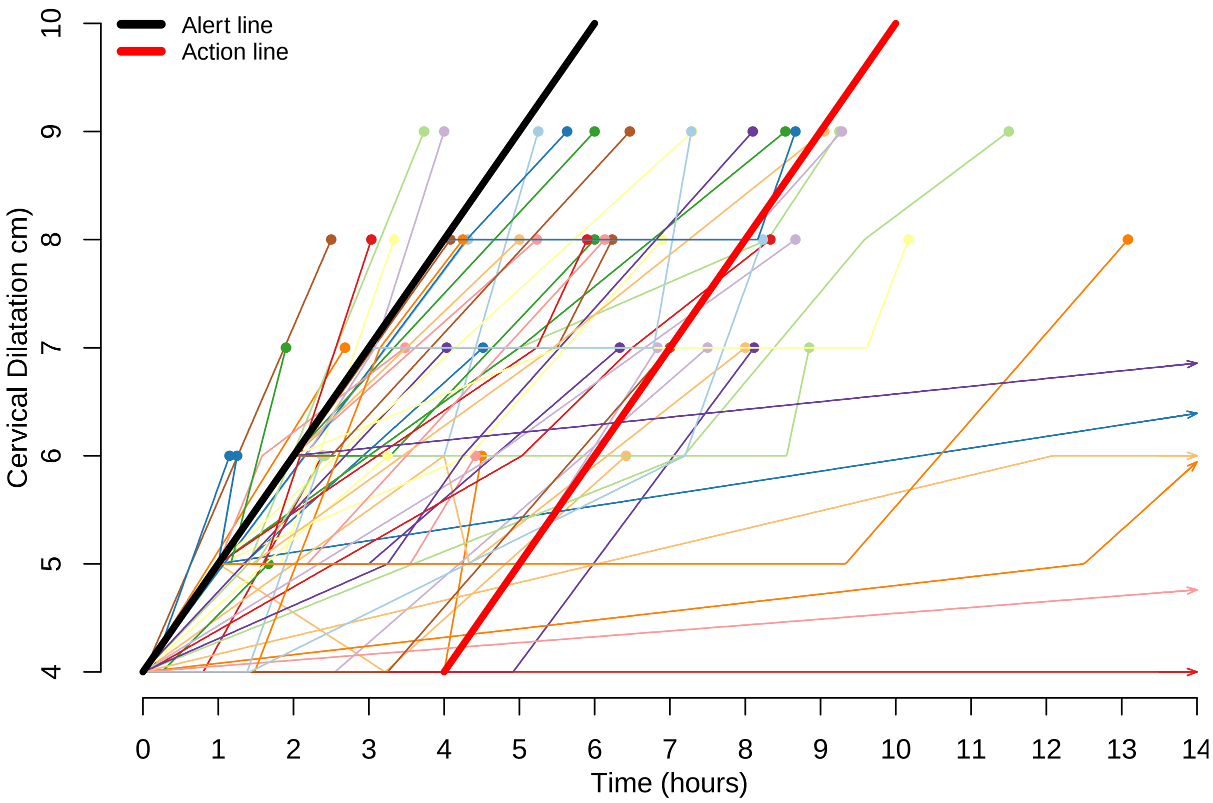

Supplement: Supplementary file 1 [file mmc1.pdf]
